# Effect of Different Fermentation Methods on the Physicochemical, Bioactive and Volatile Characteristics of Wolfberry Vinegar

**DOI:** 10.3390/foods14061078

**Published:** 2025-03-20

**Authors:** Xiao Qiang, Man Zhao, Ting Xia, Qi Wang, Junwei Yu, Changsheng Qiao, Huimin Zhang, Shiyang Lv, Yanhua Liu, Min Wang

**Affiliations:** State Key Laboratory of Food Nutrition and Safety, Key Laboratory of Industrial Fermentation Microbiology, College of Biotechnology, Tianjin University of Science and Technology, Tianjin 300457, China; qiangxiao0907@163.com (X.Q.); zhaoman1017@163.com (M.Z.); 17622979632@163.com (Q.W.); junweiyu@hotmail.com (J.Y.); qiaochangsheng@163.com (C.Q.); huiminz326@gmail.com (H.Z.); lsy050610a@163.com (S.L.); liu_yanhua@tust.edu.cn (Y.L.)

**Keywords:** wolfberry, vinegar, fermentation, phenolic compounds, amino acids, organic acids

## Abstract

Wolfberry (*Lycium barbarum* L.) as a functional food is rich in nutrients and bioactive substances. However, the fresh wolfberry is difficult to preserve, and its deep-processing products are required to improve. In the present study, single-strain fermentation vinegar (SFV) and mixed-strain fermentation vinegar (MFV) were prepared, and the physicochemical, bioactive compounds, antioxidant capacities and volatile characteristics were examined to obtain an optimal method. The results showed that reducing sugar was sufficiently utilized during mixed-strain fermentation, and more acid substances were produced compared with single-strain fermentation. Meanwhile, total phenols content (2.64 ± 0.04 mg GAE/mL), total flavonoids content (1.81 ± 0.01 mg GAE/mL) and antioxidant activities in MFV were significantly increased compared with those in SFV. Rutin, p-hydroxycinnamic acid, and 4-hydroxybenzoic acid presented higher contents in MFV than those in SFV. The contents of total organic acids (88.13 ± 0.13 mg/mL) and total amino acids (6.50 ± 0.17 mg/mL) in MFV were significantly improved compared with those in SFV. Proline, alanine and serine were the top three amino acids in MFV. Moreover, acids, eaters, and alcohols were the pre-dominant volatile organic compounds in MFV, which were higher 9.49%, 55.27%, 18.72% in MFV than those in SFV, respectively. The results suggest that MFV efficiently enhances potential health benefits and flavor, which increases the economic value of wolfberry.

## 1. Introduction

Wolfberry (*Lycium barbarum* L.) is a homology plant of medicine and food, which belongs to the *Solanaceae* family. Wolfberry contains various nutrients and bioactive substances such as polyphenols, flavonoids, and amino acids [1,2]. These components of wolfberry with the value of healthcare include antioxidants, hypoglycemic, liver protective, and anti-tumor properties [3,4,5,6]. Because of its unique nutrition and health benefits, wolfberry is favored by consumers. At present, the main products of wolfberry are dried fruits and wolfberry juice. In addition, wolfberry is difficult to store and transport, which limits its economic value. Fermentation is an effective method of biotechnology to improve the economic value and sensory characteristics of fruits [7].

Fruit vinegar is a fermented product made by two-stage fermentation processes including alcohol fermentation and acetic acid fermentation. Many nutrients in fruits are effectively extracted by fermentation, such as vitamins, dietary fiber, minerals, and carbohydrates [8]. Meanwhile, many functional components are produced during the fermentation process, including organic acids and amino acids of fruit vinegar [9]. These ingredients greatly improve the health value of fruit vinegar, such as antioxidants, anti-cancer, and blood lipids [10,11,12,13]. Therefore, wolfberry fruit is produced into wolfberry vinegar by fermentation, which will enhance its potential health benefits [9,13].

Fruit vinegar fermentation involves alcohol fermentation and acetic acid fermentation. Single strain fermentation refers to the use of only a single microbial strain during a specific fermentation stage, including only added yeast alcohol fermentation, or only added acetic acid bacteria in acetic acid fermentation [14]. Mixed strain fermentation usually refers to the simultaneous addition of a combination of two or more microbial strains at a certain fermentation stage [15]. Previous studies have shown that mixed-strain fermentation has a positive effect on flavor and bioactive components compared with single-strain fermentation. Yeast can provide many nutritional factors, including amino acids and vitamins [16]. Lactic acid bacteria can decompose large molecular substances during fermentation to produce carbon and nitrogen sources, which provide energy sources for yeast and improve aroma during alcohol fermentation [17,18]. Chen et al. reported that the total phenolic content (2.42 ± 0.11 mg GAE/mL) of citrus vinegar was higher in the mixed culture (*Saccharomyces cerevisiae* and *Lactobacillus plantarum AS1.555*) than that in the pure culture (*Saccharomyces cerevisiae*) [16]. Another study reported that the contents of total titratable acid (4.62 ± 0.07 g/dL) and vitamin C (24.50 ± 1.15 AAE mg/L) were significantly increased in mango vinegar by mixed-strain fermentation (*Saccharomyces cerevisiae* and *Lactobacillus plantarum 90*) compared with that by single-strain fermentation. In addition, mixed-strain fermentation also affected the volatile organic compounds contents, effectively improving the flavor and taste of mango vinegar [19]. In our previous study, we have found that single-strain fermentation increased the total phenolics, total flavonoids contents, and antioxidant activity [13]. However, the effects of mixed-strain fermentation on wolfberry vinegar are required to be studied further.

The aim of this study is to compare single-strain fermentation and mixed-strain fermentation methods to find strategies that can improve the added value of wolfberry products. In this study, wolfberry was used as a raw material to produce vinegar by MFV with active dry wine yeast RW, *Lactobacillus plantarum L46* and *Lactobacillus helveticus H1* in alcohol fermentation. Then, physicochemical properties, active ingredients, and volatile compounds in wolfberry vinegar were determined to compare single and mixed-strain fermentation. This study will provide novel strategies for deep-processed product of wolfberry to enhance its nutrition and economic value.

## 2. Materials and Methods

### 2.1. Materials, Strains and Chemicals

Wolfberry juice (WJ) was provided from the Ningxia Zhongning Goji Industry Innovation Research Institute (Zhongwei, China). Active dry wine yeast RW was provided by Angel Yeast Co., Ltd. (Yichang, China). *Lactobacillus plantarum L46*, *Lactobacillus helveticus H1* and *Acetobacter Pasteurianus AC2005* were isolated and preserved by the Laboratory of Systematic Microbiology and Biomanufacturing, Tianjin University of Science and Technology. Rutin and gallic acid standards were purchased from Shanghai Yuanye Biotechnology Co. Ltd. (Shanghai, China). 1,1-Diphenyl-2-picrylhydrazyl (DPPH) was purchased from Sinopharm Chemical Reagent Co., Ltd. (Shanghai, China). 2,2′-Azinobis (3-ethlybenzothiazoline)-6-sulfonic acid (ABTS, Cat No. S0119) and ferric reducing antioxidant power (FRAP, Cat No. S0116) assay kits were provided from Beyotime Institute of Biotechnology (Shanghai, China).

### 2.2. Sample Preparation

WJ (1 L) was incubated at 22 °C for 5–6 days with active dry wine yeast RW (10^6^ CFU/mL) to obtain single-strain fermentation wolfberry wine (SFW, about 850 mL). Meanwhile, WJ was fermented at 22 °C for 5–6 days with active dry wine yeast RW (10^6^ CFU/mL), *Lactobacillus plantarum L46* (10^6^ CFU/mL), and *Lactobacillus helveticus H1* (10^6^ CFU/mL) to obtain mixed-strain fermentation wolfberry wine (MFW, about 850 mL). The alcohol fermentation ended when the alcohol content no longer increased. Then, SFW and MFW were centrifuged and inoculated with *Acetobacter Pasteurianus AC2005* (10^6^–10^7^ CFU/mL) at 30 °C and 180 rpm for 6 days, respectively. The fermentation ended when the acetic acid content was stabilized. The SFV and MFV were obtained. To reduce the batch-to-batch variation in SFV and MFV, three batches of wolfberry fruits were collected in the same year.

### 2.3. Physicochemical Analysis

The pH values of SFV and MFV were measured by the pH meter (TecFront Electronics Co. Ltd., Shanghai, China) [13]. The soluble solids were determined by a hand-held refractometer (Aitako PR-101R, Tokyo, Japan). Total acidity was determined according to GB/T 12456-2008 [20]. Briefly, according to the principle of acid-base neutralization, the acid in the test solution was titrated with alkaline solution and phenolphthalein was used as an indicator to determine the titration endpoint. The total acid content was calculated based on the consumption of the alkaline solution. Reducing sugar was determined according to GB 5009.7-2016 [21]. Briefly, methylene blue was used as an indicator to titrate the alkaline copper tartrate solution under heating conditions. The reducing sugar content was calculated based on the volume consumed by the sample solution. Lactic acid was determined according to GB 2023-2003 [22]. Briefly, the sample with sodium hydroxide solution was added in phenolphthalein indicator, and finally sulfuric acid solution was added for titration. The alcoholic concentration of the fermented product was determined according to GB 5009.225-2016 [23] with a hydrometer (Aoqiluo Pu Trading Co., Ltd., Tianjin, China).

### 2.4. Determination of Total Phenols and Total Flavonoids

The total phenols content (TPC) was determined by Folin–Ciocalteu method [17]. The total flavonoids content (TFC) in *Lycium barbarum* vinegar was determined the colorimetric assay technique [24]. The absorbance was measured by UV-765 spectrophotometer (Duyang Precision Instrument Co., Ltd., Shanghai, China). The data of TPC and TFC were presented with gallic acid equivalents (mg GAE/mL) and rutin equivalents (mg RE/mL) as content units, respectively.

### 2.5. Determination of Antioxidant Activity In Vitro

The 1,1-diphenyl-2-picrylhydrazyl (DPPH) assay, 2,2’-Azinobis-(3-ethylbenzthiazoline-6-sulphonate) (ABTS) assay, and ferric reducing antioxidant power (FRAP) assay were detected with the methods utilized before [24]. Briefly, In the DPPH assay, the samples were added 20 µL in 96-well plates with DPPH solution (180 µL). After 30 min at room temperature, the samples were measured by microplate readers to read the absorbance at 517 nm. In the ABTS assay, the samples (10 µL) and ABTS^+^ radical cation solution (200 µL) were combined and incubated for 6 min. The samples were determined using a microplate reader to detect the absorbance at 734 nm. In the FRAP assay, the samples (5 µL) and FRAP solution (180 µL) were incubated at 37 °C for 3–5 min. The samples were detected using a microplate reader to measure the absorbance at 593 nm.

### 2.6. Determination of Phenolic Compounds

The phenolic compounds were analyzed by UPLC-Q Exactive-MS/MS (Thermo, Waltham, MA, USA) equipped with a column of HSS-T3 (50 mm × 2.1 mm, 1.8 µm, Waters, Milford, MA, USA) [25]. The samples were centrifuged (12,000 rpm) at 4 °C for 10 min. Furthermore, the supernatant was filtered through a 0.22 µm PES membrane filter and then injected into samplers with 2 µL. The detection wavelength was 278 nm. The mobile phase composed of 1% of formic acid in water (solvent A) and 1% of formic acid in acetonitrile (solvent B) was set as following gradient program: 0–6 min, 10% B; 6–9.1 min, 60% B; 9.1–12 min, 10% B. Column oven temperature was 40 °C. For the mass spectrometer analysis, the ion source was an ESI ionization source with negative ion mode; ion spray voltage was −2800 V; ion source temperature was 350 °C; ion transfer tube temperature was 320 °C. The qualitative analysis of phenolic compounds was performed according to the retention time, retention index and mass spectrum information of the substance.

### 2.7. Determination of Organic Acids

The determination of organic acids was performed according to a previous method, with minor modification [26]. Briefly, samples were filtered with a 0.22 µm microporous membrane to effectively remove impurities. A high-performance liquid chromatography (HPLC, Agilent, New York, NY, USA) system with an Aminex HPX-87H 300 × 7.8 (mm) column (Bio-Rad, Hercules, CA, USA) was used for organic acid analysis. Then, the sample was analyzed under the following mobile phase was 5 mmoL/L H_2_SO_4_. The flow rate was 0.6 mL/min, and injection volume was 20 µL. The UV detector wavelength was 215 nm, and the column temperature was 30 °C.

### 2.8. Determination of Amino Acids

The amino acids contents of SFV and MFV were analyzed by amino acid analyzer (Biochrom, Cambridge, UK) with a LCAK06/Na (4.6 mm × 150 mm) column based on the previous study with slight modifications [24]. The mobile phase was hydrochloric acid buffer with 0.45 mL/min flow rate The column temperature was 30–70 °C, Ex 440 nm, Em 570 nm. The amino acids were derivatized by ninhydrin reagents at 130 °C for 60 min.

### 2.9. Determination of Volatile Organic Compounds

The volatile organic compounds (VOCs) were analyzed by HS-SPME-GC-MS (Thermo, Waltham, MA, USA) equipped with a column of HP-5 (30 m × 0.25 mm, 0.25 µm) [27]. 2 mL sample and 2 g NaCl were pre-equilibrated, and then the SPME fiber was inserted into the head space and maintained for 40 min at 60 °C. The GC oven temperature was initially 40 °C, and increased to 150 °C at 4 °C/min and held constant for 1 min, and then rose to 250 °C at 8 °C/min and held constant for 6 min. Helium was used as the carrier gas at a flow rate of 1.0 mL/min. The ion source was set at 250 °C. The mass-to-charge ratio was 35–500 m/z, and the solvent elution delay time was 1.5 min.

### 2.10. Statistical Analysis

The data were given as mean ± standard deviation (S.D.), each set of experimental data was repeated three times. Statistical analysis was performed using the GraphPad Prism 8.0.1 software (GraphPad Software Inc., San Diego, CA, USA), and SPSS 20 for Windows (SPSS Inc., Chicago, IL, USA). Clustering heat map and Principal component analysis (PCA) was conducted with Origin 8.0 software. Pearson’s correlation test was applied to the related correlation analyses. *p* < 0.05 indicate a significant difference.

## 3. Results and Discussion

### 3.1. Physicochemical Property of Wolfberry Vinegar

**Figure 1:**
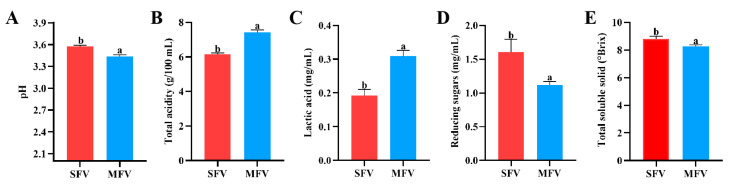
Physical and chemical indexes in SFV and MFV. (**A**) pH, (**B**) total acidity, (**C**) lactic acid, (**D**) reducing sugar, (**E**) total soluble solids. a, b present statistically significant differences (*p* < 0.05). SFV: single-strain fermentation vinegar; MFV: mixed-strain fermentation vinegar.

The physical and chemical characteristics were analyzed in MFV and SFV. As shown in Figure 1A, the pH value of MFV reached 3.44, which was significantly lower than that in SFV. Total acidity is an important parameter to measure the fermentation state and product quality of wolfberry vinegar [28]. In Figure 1B, total acidity was significantly increased to 7.42 ± 0.12 g/100 mL in MFV compared with that in SFV. This is because of the continuous production of acid during the process of fermentation, and the evolution of acetic acid during acetic acid fermentation is negatively linear with pH [29,30]. In Figure 1C, the lactic acid content in MFV was 0.309 ± 0.014 mg/mL, which was higher than that in SFV (*p* < 0.05). The reason for the increase in lactic acid in MFV was that the lactic acid bacteria strains used produce lactic acid during alcohol fermentation process [31]. In addition, in Figure 1D,E, the content of reducing sugar in MFV was 1.12 ± 0.05 mg/mL, which was significantly lower than that in SFV (1.60 ± 0.16 mg/mL). Total soluble solids are also important indicators to evaluate the quality of vinegar [13]. Total soluble solids in MFV (8.27 ± 0.094 °Brix) were notably lower than that in SFV. Some studies reported that the content of reducing sugar and total soluble solids were significantly decreased in fermented prunus and rose fruit vinegars, which supported our research results [29,32]. The reduction in reducing sugar is due to the fact that the sugar is consumed and converted to alcohol and acetic acid during the fermentation processes [33]. In conclusion, mixed-strain fermentation can more fully utilize reducing sugar, and produce more acid substances.

### 3.2. TPC, TFC and Antioxidant Capacities of Wolfberry Vinegar

As shown in Table 1, TPC was 2.99 ± 0.05 mg GAE/mL in MFV, which was significantly higher than that in SFV (2.64 ± 0.04 mg GAE/mL). Meanwhile, TFC in MFV was 1.81 ± 0.01 mg GAE/mL, which was significantly higher than that in SFV (1.21 ± 0.06 mg GAE/mL) (*p* < 0.05). Some studies had reported that TPC and TFC in fruit juice were significantly increased after fermentation with lactic acid bacteria [34,35]. Wang et al. found that TPC and TFC in fruit juice were increased after lactic acid bacteria fermentation, which was attributed that lactic acid bacteria deglycosylase more glycosylated phenolics of the fruit juice during the fermentation, and then release simpler phenolics and flavanols compounds from plant cells [36]. These results suggested that more TPC and TFC in MFV than those in SFV partly due to the mixed culture fermentation added lactic acid bacteria.

It has been reported that phenolic compounds, as reducing agents and free-radical scavengers, possessed remarkable antioxidant capacities mainly attributed to the hydrogen atom transfer or electron donation to free radicals [8]. In this study, antioxidant capacities of MFV measured by DPPH, ABTS and FRAP assays were increased by 18.34%, 12.25%, and 31.59%, respectively, compared with those of SFV. Wu et al. found that the radical scavenging capacity of blackberry and blueberry juices was increased by 53.3% and 64.0%, respectively, after being fermented with L. *plantarum* through the ABTS assay [37]. It has been reported that lactic acid bacteria contribute to the degradation of complex phenolics of dietary fiber and the release of free phenolics in purple sweet potato juice, resulting in higher antioxidant activity after fermentation [36]. Therefore, the results suggested that mixed-strain fermentation increased TPC and TFC as well as improving antioxidant capacity. Mixed-strain fermentation of wolfberry vinegar preserved the active ingredients and enhanced functional activity, providing added value for high-quality wolfberry products with more health benefits.

### 3.3. Phenolic Compounds of Wolfberry Vinegar

A total of 22 phenolic compounds were detected in MFV and SFV after fermentation (Table 2). The total contents of phenolic compounds were 19.21% higher in MFV than those in SFV. Recent study reported that phenolic contents were increased in jujube-wolfberry composite juice fermented by lactic acid bacteria, which supported the higher phenolic contents in MFV [38]. The main phenolic compounds were rutin, p-hydroxycinnamic acid, and 4-hydroxybenzoic acid in MFV and SFV. In particular, the concentration of rutin (5457.97 ± 450.30 ng/L) was highest in MFV, which was 21.3% higher than that in SFV. Rutin is a common dietary flavone, possessing antibacterial, anti-inflammatory, anti-cancer, and anti-diabetic effects [39,40]. Liu et al. reported that *Lactobacillus plantarum* fermentation led to a remarkable increase in rutin in wolfberry pulp, which explain rutin content is higher after mixed fermentation by active dry wine yeast RW, *L*. *plantarum L46* and *L. helveticus H1* [41]. Additionally, the contents of p-hydroxycinnamic acid and 4-hydroxybenzoic acid in MFV were increased by 14.5% and 25.0%, respectively, compared with those in SFV. The content of caffeic acid was 7.25 times higher in MFV than that in SFV. The increased content of caffeic acid in MFV could be due to the hydrolysis of cinnamoyl glucoside and thiocyanate [38]. The content of catechin in MFV was significantly lower than that in SFV, which is potentially due to the degradation or transformation of catechins by lactic acid bacteria during fermentation through de-glycosylate, de-esterify, de-carboxylate and de-methylate [42]. These results indicated that mixed-strain fermentation could enhance the phenolic composition.

### 3.4. The Relationship Between Phenolic Compounds and Antioxidant Activities of MFV

**Figure 2:**
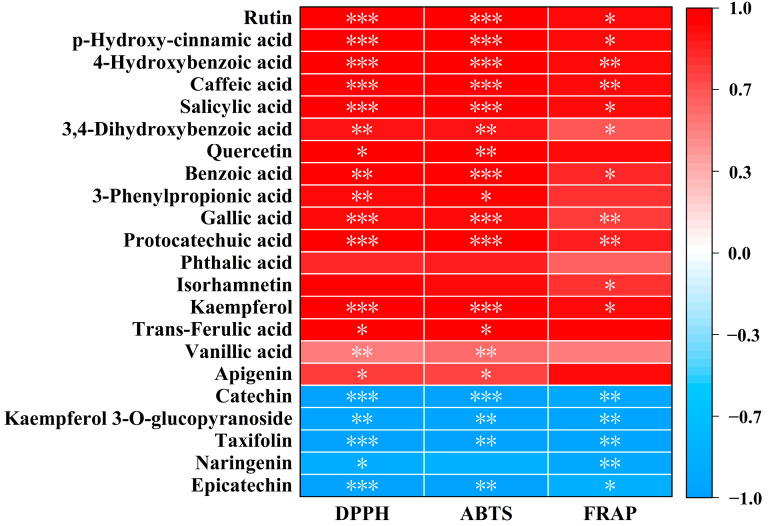
Correlation analysis of antioxidant activity and phenolic compounds of single-strain fermentation vinegar and mixed-strain fermentation vinegar. * *p* < 0.05, ** *p* < 0.01, *** *p* < 0.001 reflect the significance of activity in vitro.

It has been reported that the antioxidant activity of polyphenolics is closely related to their chemical structure, which is significantly influenced by base-linked groups [37]. Pearson’s correlation analysis of antioxidant activities and phenolic compounds was shown in Figure 2. The antioxidant activities were positively correlated with flavonoids (rutin, kaempferol), cinnamic acid derivatives (p-hydroxycinnamic acid, caffeic acid), benzoic acid derivatives (4-hydroxybenzoic acid, 3,4-dihydroxybenzoic acid, benzoic acid, gallic acid, protocatechuic acid), and salicylic acid. Rutin, p-hydroxycinnamic acid, 4-hydroxybenzoic acid, caffeic acid, and salicylic acid were the five most abundant acids in MFV, which were significantly higher in MFV than those in SFV. However, the antioxidant activities were negatively correlated with the contents of catechin, kaempferol 3-o-glucopyranoside, taxifolin, and epicatechin, which were significantly lower in MFV than those in SFV (Table 2). These results indicated that the increased antioxidant activity of MFV should be mainly attributed to the increased contents of rutin, p-hydroxy-cinnamic acid, 4-hydroxybenzoic acid, caffeic acid and salicylic acid. Wang et al. reported that vanillic acid, protocatechuic acid and caffeic acid in purple sweet potato juice fermented by L. *plantarum* were positively correlated with DPPH and ABTS radical scavenging activity, but peonidin-3-o-glucoside was negatively correlated with antioxidant activities [36]. The contents of these phenolics positively correlated with antioxidant activities were significantly increased, while peonidin-3-o-glucoside was decreased after fermentation [36]. These results indicated that the antioxidant activities of MFV were mainly attributed to the higher phenolics through mixed-strain fermentation compared with single-strain fermentation.

### 3.5. Organic Acids of Wolfberry Vinegar

Organic acids are vital components contributing to vinegar flavor, and the composition and concentration also influence the stability, organoleptic properties and nutrition quality of vinegar [43]. The organic acids contents in SFV and MFV were shown in Table 3. There are nine organic acids detected in SFV and MFV. The total organic acids contents of MFV were 88.13 ± 0.13 mg/mL, which were significantly higher than those of SFV (81.72 ± 0.12 mg/mL). Acetic acid, citric acid and malic acid were the primary organic acids. Acetic acid was the most abundant organic acids, accounting for more than 70% of the total organic acids in both SFV and MFV. The main reason for high acetic acid content was the oxidation of alcohol to acetic acid by acetic acid bacteria [44]. Acetic acid (70.92 ± 0.12 mg/mL) in MFV was significantly higher than that in SFV (64.85 ± 0.20 mg/mL). Citric acid and malic acid in MFV were 5.05 ± 0.03 mg/mL and 4.98 ± 0.06 mg/mL, respectively, which were lower than those in SFV (5.16 ± 0.03 mg/mL, 5.14 ± 0.05 mg/mL) (*p* > 0.05). However, lactic acid (1.47 ± 0.02 mg/mL) in MFV was higher than that in SFV (1.38 ± 0.07 mg/mL). The reason for the decreased content of citric and malic acid and the increased content of lactic acid in MFV is that citric acid and malic acid can be converted into lactic acid by lactic acid bacteria [45,46]. The content of tartaric acid (1.62 ± 0.02 mg/mL) in MFV was significantly higher than that in SFV (1.34 ± 0.03 mg/mL). It has been reported that tartaric acid assisted in maintaining acidity, lowering pH, inhibiting bacterial growth, and preserving long-term freshness [47]. Ji et al. reported that the content of tartaric acid was increased in apple juices fermented with lactic acid bacteria, which supported our results [48]. The above results indicated that mixed-strain fermentation enhanced the total contents of organic acids in MFV, especially acetic acid and tartaric acid.

### 3.6. Comparison of Amino Acid Between MFV and SFV

As shown in Table 4, a total of 17 amino acids were detected by amino acid analyzer in SFV and MFV, which including 6 sweet amino acids, 8 bitter amino acids, 2 umami amino acids, and 1 tasteless amino acid. The total amino acids contents of MFV were 6.50 ± 0.17 mg/mL, which were significantly higher than those of SFV (4.43 ± 0.12 mg/mL) (*p* < 0.05). Proline (1.936 ± 0.173 mg/mL) was the highest in sweet amino acids of MFV, followed by alanine (1.138 ± 0.108 mg/mL) and serine (0.743 ± 0.022 mg/mL). Sweet amino acids were higher in MFV than that of SFV. A previous study has reported that proline was also the most abundant amino acid in Modena Vinegar (BVM), which was in line with our results [49]. The presence of arginine, which accounts for bitter amino acids, was significantly reduced to 0.048 mg/L in MFV that compared to SFV (*p* < 0.05). Meanwhile, glutamic acid was the predominant umami amino acid in SFV and MFV, and the glutamic acid contents (0.309 ± 0.018 mg/mL) were higher than those in MFV (*p* < 0.05). In addition, the contents of total essential amino acids were 1.835 ± 0.186 mg/mL in MFV, which were higher than those in SFV (*p* < 0.05). Chen et al. reported that the amino acid content of citrus vinegar in mixed cultures of *Saccharomyces cerevisiae* and *Lactobacillus plantarum* (3229 ± 23.3 mg/L) was significantly higher than pure culture of *Saccharomyces cerevisiae* (3144 ± 22.5 mg/L) (*p* < 0.05), making the taste of citrus vinegar stronger and more umami [16]. It has been reported that certain proteins and peptides were decomposed by lactic acid bacteria during the fermentation process, which led to the increase in amino acids [50]. The above results indicated that mixed cultures enhanced the total content of free amino acids in MFV, particularly sweet and essential amino acids enhanced the flavor and nutritional value of wolfberry vinegar.

### 3.7. Volatile Organic Compounds of Wolfberry Vinegar

**Figure 3:**
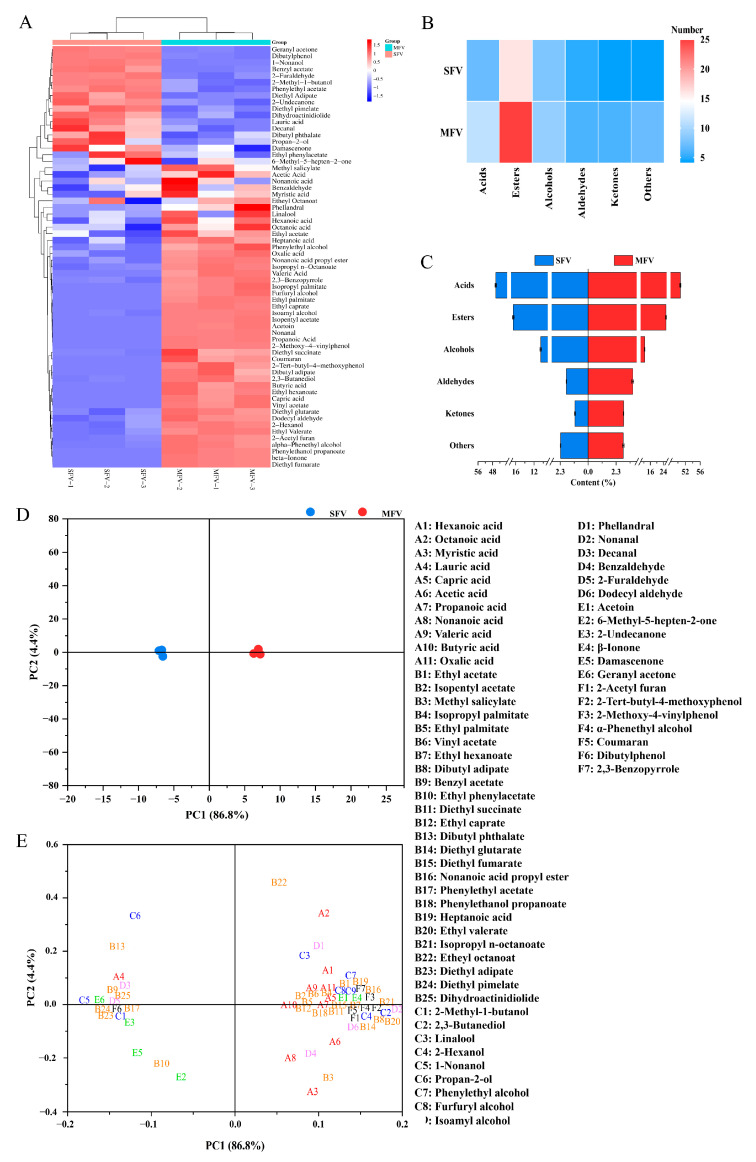
(**A**) Clustering heat map, (**B**) number, (**C**) relative content of volatile organic compounds in SFV and MFV. (**D**) Principal component analysis (PCA) and (**E**) loadings scatter plot of volatile organic compounds in SFV and MFV. The consecutive numbers A1 to F7 on the right side of the figure represent the names of volatile compounds (A: acid, B: ester, C: alcohol, D: aldehyde, E: ketone, F: others). SFV: single-strain fermentation vinegar; MFV: mixed-strain fermentation vinegar.

Volatile organic compounds (VOCs) are the important indicators to evaluate the quality of fruit vinegar and affect consumer preferences, which play a decisive role in fruit vinegar taste presentation. Significant differences were observed in the types and contents of VOCs between SFV and MFV. A total of 44 and 64 VOCs were found in SFV and MFV, respectively (Figure 3A). In total, 11 acids, 25 esters, 9 alcohols, 6 aldehydes, 6 ketones, and 7 others were detected in MFV, their relative contents were 50.664%, 25.646%, 12.509%, 3.672%, 2.914%, and 2.892%, respectively (Figure 3B,C). Acids were the main source of vinegar flavor, with the highest content in VOCs. The contents of acids were significantly higher in MFV than those in SFV (*p* < 0.05), and acetic acid was the predominant acid (43.13%) (Table 5). It has been reported that acetic acid bacteria produce a large amount of acetic acid and other organic acids through fermentation [24]. In addition, lactic acid bacteria also consume residual sugar to increase acetic acid concentration [51]. These may be the principal reason for the higher acid contents in MFV. The second largest proportion of VOCs in SFV and MFV were esters, and the total esters of MCCV (25.646%) were 1.55-fold higher than that of SFV (16.517%). Meanwhile, esters were the most diverse type among VOCs. Phenylethyl acetate contents were the highest, followed by diethyl succinate and ethyl caprate in MFV. Previous study has reported that the addition of *L. plantarum* to the fermentation process increased the production of ester compounds [52].

Moreover, the unique odor of fruit vinegar is partly attributed to its volatile alcohols [53]. The contents of total alcohol, phenylethyl alcohol and Isoamyl alcohol were significantly higher in MFV than that in SFV (*p* < 0.05). Chen et al. reported that the total alcohols and phenylethyl alcohol concentrations of citrus vinegar by mixed bacterial fermentation were significantly higher than those by single bacterial fermentation [16]. The increased alcohols effectively improved the flavor and quality of citrus vinegar. Aldehydes and ketones in VOCs are oxidized to acids or reduced to alcohols by lactic acid bacteria [28]. The contents of aldehydes and ketones were significantly increased in MFV compared with those in SFV (*p* < 0.05). Nonanal was the main aldehydes, and acetoin was the primary ketones in MFV. Therefore, the presence of VOCs gives vinegar a soft taste and unique flavor.

The impact of mixed bacterial fermentation on the VOCs was visually analyzed by principal component analysis (PCA). As shown in Figure 3D, the VOCs showed significant differences between SFV and MFV. The cumulative variance explained by PC1 and PC2 was 91.2%, of which PC1 accounted for 86.8% and PC2 accounted for 4.4% of the data variance. In addition, PCA was conducted on 64 compounds to better explain the effects of various VOCs in SFV and MFV (Figure 3E). The results indicated that acids, esters, and alcohols mainly contributed to MFV, while esters and ketones contributed to SFV.

## 4. Conclusions

This study demonstrated that mixing with active dry wine yeast RW, *Lactobacillus plantarum L46* and *Lactobacillus helveticus H1* during alcohol fermentation can significantly improve the quality and flavor of wolfberry vinegar. The pH value of MFV was decreased, and the contents of total acid and lactic acid were increased. TPC, TFC, and antioxidant activities in MFV were significantly increased by 11.71%, 33.15%, 12.25–31.59%, respectively, compared with those in SFV, which endowed mixed-strain fermented wolfberry vinegar with potential health benefits. Meanwhile, the total organic acid and amino acid contents in MFV were 7.27% and 31.85% higher than those in SFV, which contributed to better flavor and nutritional value of MFV. In addition, the contents of volatile organic compounds were significantly increased in MFV compared to SFV, which enhanced the sensory quality of vinegar. This study presented a novel strategy to produce high-quality wolfberry vinegar with more bioactive and flavored substances, which enhancing both health benefits and commercial potential. Future research should focus on improving the production efficiency of wolfberry vinegar to facilitate the expansion of production. And further functional experiments in vivo will be conducted, which can provide more theoretical basis for market application.

## Figures and Tables

**Table 1:** TPC, TFC and antioxidant capacities of wolfberry vinegar.

	Indexes	Content	
		SFV	MFV
Active ingredients	TPC (mg GAE/mL)	2.64 ± 0.04 b	2.99 ± 0.05 a
	TFC (mg RE/mL)	1.21 ± 0.06 b	1.81 ± 0.01 a
Antioxidant activities	DPPH (mM Trolox/L)	17.61 ± 0.11 b	20.84 ± 0.13 a
	ABTS (mM Trolox/L)	16.21 ± 0.43 b	18.67 ± 0.07 a
	FRAP (mM Trolox/L)	4.40 ± 0.23 b	5.79 ± 0.26 a

SFV: single-strain fermentation vinegar; MFV: mixed-strain fermentation vinegar; TPC: Total phenols content; TFC: total flavonoids content; DPPH: 1,1-Diphenyl-2-picrylhydrazyl; ABTS: 2,2′-Azinobis (3-ethlybenzothiazoline)-6-sulfonic acid; FRAP: ferric reducing antioxidant power. Data are presented as mean ± standard deviation of three sets of replicate experiments. Values in the same row with different letters are significantly different at *p* < 0.05.

**Table 2:** Contents of phenolic compounds in SFV and MFV (ng/L).

Phenolics	Retention Time (min)	SFV	MFV
Protocatechuic acid	3.24	23.57 ± 2.48 b	36.07 ± 1.20 a
Catechin	3.97	68.41 ± 2.55 a	14.61 ± 0.25 b
Vanillic acid	4.20	2.08 ± 0.65 a	2.46 ± 0.33 a
Salicylic acid	5.72	116.86 ± 7.36 b	420.92 ± 8.66 a
Gallic acid	5.99	39.19 ±4.43 b	49.84 ±3.04 a
Benzoic acid	6.04	57.91 ± 7.23 b	80.59 ± 2.19 a
Kaempferol 3-Oglucopyranoside	6.79	7.53 ± 0.22 a	6.22 ± 0.49 b
Quercetin	7.09	67.50 ± 1.80 b	113.77 ± 0.89 a
Apigenin	7.59	0.11 ± 0.01 b	0.20 ± 0.09 a
Isorhamnetin	7.74	2.36 ± 0.33 b	11.42 ± 0.17 a
Trans-Ferulic acid	9.69	3.51 ± 0.02 b	5.31 ± 0.21 a
3-Phenylpropionic acid	11.90	3.91 ± 0.57 b	56.04 ± 1.92 a
4-Hydroxybenzoic acid	13.43	579.90 ± 24.37 b	724.86 ± 56.12 a
Epicatechin	14.82	1.07 ± 0.01 a	0.55 ± 0.09 b
p-Hydroxy-cinnamic acid	17.15	1706.41 ± 82.69 b	1954.46 ± 59.74 a
Kaempferol	17.67	0.82 ± 0.12 b	8.06 ± 0.09 a
Naringenin	19.59	0.86 ± 0.11 a	0.69 ± 0.05 b
Taxifolin	21.69	2.45 ± 0.10 a	1.07 ± 0.09 b
Phthalic acid	23.31	19.44 ± 3.72 b	25.74 ± 2.96 a
Caffeic acid	24.35	72.38 ± 0.77 b	525.01 ± 1.06 a
Protocatechuic acid	26.04	111.90 ± 8.30 b	202.46 ± 0.21 a
Rutin	35.61	4501.29 ± 384.81 b	5457.97 ± 450.30 a
Total contents		7389.46 ± 483.44 b	9698.31 ± 571.20 a

SFV: single-strain fermentation vinegar; MFV: mixed-strain fermentation vinegar. Data are presented as mean ± standard deviation of three sets of replicate experiments. Values in the same row with different letters are significantly different at *p* < 0.05.

**Table 3:** Contents of organic acids in SFV and MFV (mg/mL).

Organic Acids	Retention Time (min)	SFV	MFV
Tartaric acid	6.03	1.34 ± 0.03 b	1.62 ± 0.02 a
Oxalic acid	6.74	0.95 ± 0.02 a	1.01 ± 0.02 a
Citric acid	7.76	5.16 ± 0.03 a	5.05 ± 0.03 a
Ascorbic acid	8.20	0.36 ± 0.02 a	0.44 ± 0.04 a
Malic acid	9.36	5.14 ± 0.05 a	4.98 ± 0.06 a
Acetic Acid	9.89	64.85 ± 0.20 b	70.92 ± 0.12 a
Lactic acid	11.16	1.38 ± 0.07 a	1.47 ± 0.02 a
Succinic acid	12.72	1.25 ± 0.06 a	1.30 ± 0.06 a
Fumaric acid	15.72	1.29 ± 0.03 a	1.34 ± 0.04 a
Total organic acids		81.72 ± 0.12 b	88.13 ± 0.13 a

SFV: single-strain fermentation vinegar; MFV: mixed-strain fermentation vinegar. Data are presented as mean ± standard deviation of three sets of replicate experiments. Values in the same row with different letters are significantly different at *p* < 0.05.

**Table 4:** Contents of amino acids in SFV and MFV.

Amino Acids	Taste Attributes	Concentration (mg/mL)	
		SFV	MFV
Proline	sweet	1.595 ± 0.386 b	1.936 ± 0.173 a
Alanine	sweet	0.716 ± 0.523 b	1.138 ± 0.108 a
Serine	sweet	0.397 ± 0.232 b	0.743 ± 0.022 a
Threonine	sweet	0.204 ± 0.069 b	0.349 ± 0.015 a
Glycine	sweet	0.088 ± 0.018 a	0.117 ± 0.011 a
Histidine	sweet	0.028 ± 0.010 a	0.043 ± 0.017 a
Lysine	sweet and bitter	0.123 ± 0.025 b	0.890 ± 0.042 a
Arginine	bitter	0.464 ± 0.165 a	0.048 ± 0.007 b
Methionine	bitter	0.071 ± 0.063 a	0.107 ± 0.015 a
Isoleucine	bitter	0.094 ± 0.069 b	0.148 ± 0.024 a
Leucine	bitter	0.066 ± 0.023 b	0.121 ± 0.027 a
Valine	bitter	0.054 ± 0.026 a	0.082 ± 0.013 a
Phenylalanine	bitter	0.098 ± 0.030 a	0.140 ± 0.057 a
Tyrosine	bitter	0.087 ± 0.036 a	0.105 ± 0.047 a
Aspartic acid	umami	0.015 ± 0.003 a	0.051 ± 0.029 a
Glutamic acid	umami	0.216 ± 0.083 b	0.309 ± 0.018 a
Cysteine	tasteless	0.117 ± 0.095 b	0.178 ± 0.006 a
Total essential free amino acids		0.708 ± 0.217 b	1.835 ± 0.186 a
Content of total free amino acids		4.43 ± 0.12 b	6.50 ± 0.17 a

SFV: single-strain fermentation vinegar; MFV: mixed-strain fermentation vinegar. Each value was expressed as mean ± S.D. (n = 3); Values in the same row with different letters are significantly different at *p* < 0.05.

**Table 5:** Volatile organic compounds in SFV and MFV.

Class	Compounds	Relative Content (%)	
		SFV	MFV
Acids	Hexanoic acid	0.596 ± 0.127 b	0.682 ± 0.133 a
	Octanoic acid	1.593 ± 0.220 b	2.17 ± 0.994 a
	Myristic acid	1.035 ± 0.381 a	1.263 ± 0.373 a
	Lauric acid	0.489 ± 0.097 a	0.159 ± 0.112 b
	Nonanoic acid	0.593 ± 0.182 b	0.703 ± 0.347 a
	Capric acid	-	0.822 ± 0.427 a
	Acetic Acid	41.673 ± 0.623 b	43.13 ± 0.513 a
	Propanoic Acid	-	0.892 ± 0.276 a
	Valeric Acid	-	0.13 ± 0.082 a
	Butyric acid	-	0.235 ± 0.109 a
	Oxalic acid	0.294 ± 0.120 b	0.477 ± 0.095 a
	Total	46.273 ± 0.330 b	50.664 ± 0.132 a
Esters	Ethyl acetate	0.536 ± 0.068 b	0.650 ± 0.131 a
	Isopentyl acetate	-	0.834 ± 0.279 a
	Methyl salicylate	0.356 ± 0.069 b	0.469 ± 0.159 a
	Isopropyl palmitate	-	0.268 ± 0.093 a
	Ethyl palmitate	-	0.353 ± 0.199 a
	Dibutyl adipate	-	0.157 ± 0.096 a
	Benzyl acetate	0.417 ± 0.106 a	0.137 ± 0.102 b
	Ethyl phenylacetate	0.270 ± 0.114 a	0.239 ± 0.163 a
	Dihydroactinidiolide	0.657 ± 0.067 a	0.167 ± 0.076 b
	Ethyl hexanoate	-	0.340 ± 0.129 a
	Vinyl acetate	-	0.369 ± 0.221 a
	Diethyl succinate	0.572 ± 0.182 b	1.373 ± 0.474 a
	Ethyl caprate	-	6.300 ± 0.151 a
	Dibutyl phthalate	0.426 ± 0.197 a	0.350 ± 0.022 b
	Diethyl glutarate	0.353 ± 0.267 b	0.670 ± 0.465 a
	Etheyl Octanoat	0.238 ± 0.218 a	0.254 ± 0.263 a
	Nonanoic acid propyl ester	0.337 ± 0.114 b	0.530 ± 0.091 a
	phenylethyl acetate	9.882 ± 2.045 a	8.656 ± 2.361 b
	Phenylethanol propanoate	-	0.286 ± 0.198 a
	Heptanoic acid	0.448 ± 0.278 b	0.580 ± 0.252 a
	Ethyl Valerate	0.212 ± 0.100 b	0.514 ± 0.086 a
	Isopropyl n-Octanoate	0.442 ± 0.286 b	0.765 ± 0.420 a
	Diethyl Adipate	0.662 ± 0.075 a	0.461 ± 0.094 b
	Diethyl pimelate	0.71 ± 0.294 a	0.461 ± 0.149 b
	Diethyl fumarate	-	0.465 ± 0.268 a
	Total	16.517 ± 0.141 b	25.646 ± 0.260 a
Alcohols	2-Hexanol	0.278 ± 0.218 b	0.491 ± 0.195 a
	2-Methyl-1-butanol	0.808 ± 0.264 a	0.559 ± 0.282 b
	Linalool	0.128 ± 0.058 a	0.160 ± 0.080 a
	1-Nonanol	0.840 ± 0.435 a	0.157 ± 0.537 b
	Isoamyl alcohol	1.175 ± 0.124 b	2.498 ± 0.666 a
	2,3-Butanediol	0.180 ± 0.006 b	0.451 ± 0.099 a
	Phenylethyl alcohol	6.873 ± 2.027 b	7.721 ± 2.021 a
	1-Nonanol	0.254 ± 0.108 a	0.206 ± 0.010 a
	Furfuryl alcohol	-	0.266 ± 0.034 a
	Total	10.537 ± 0.170 b	12.509 ± 0.137 a
Aldehydes	Nonanal	-	1.864 ± 0.616 a
	2-Furaldehyde	0.492 ± 0.347 a	0.358 ± 0.181 b
	Decanal	0.368 ± 0.251 a	0.318 ± 0.180 b
	Benzaldehyde	0.347 ± 0.107 a	0.363 ± 0.216 a
	Phellandral	0.208 ± 0.100 b	0.301 ± 0.110 a
	Dodecyl aldehyde	0.373 ± 0.181 b	0.468 ± 0.269 a
	Total	1.789 ± 1.589 b	3.672 ± 0.470 a
Ketones	6-Methyl-5-hepten-2-one	0.168 ± 0.062 a	0.155 ± 0.089 a
	2-Undecanone	0.184 ± 0.077 a	0.105 ± 0.051 b
	Damascenone	0.168 ± 0.080 a	0.125 ± 0.094 a
	Geranyl acetone	0.567 ± 0.334 a	0.112 ± 0.017 b
	β-Ionone	-	0.346 ± 0.265 a
	Acetoin	-	2.070 ± 0.651 a
	Total	1.087 ± 1.005 b	2.914 ± 1.130 a
Others	Dibutylphenol	1.224 ± 0.054 a	0.344 ± 0.054 b
	α-Phenethyl alcohol	0.535 ± 0.005 b	0.768 ± 0.054 a
	2-Methoxy-4-vinylphenol	-	0.516 ± 0.054 a
	2-Acetyl furan	0.135 ± 0.010 b	0.353 ± 0.054 a
	2,3-Benzopyrrole	0.375 ± 0.054 b	0.603 ± 0.054 a
	2-Tert-butyl-4-methoxyphenol	-	0.149 ± 0.054 a
	Coumaran	-	0.157 ± 0.054 a
	Total	2.269 ± 0.054 b	2.892 ± 0.054 a

SFV: single-strain fermentation vinegar; MFV: mixed-strain fermentation vinegar. Each value was expressed as mean ± S.D. (n = 3); Values in the same row with different letters are significantly different at *p* < 0.05; -: not detected.

## Data Availability

The original contributions presented in this study are included in the article. Further inquiries can be directed to the corresponding author.
